# *Atolla reynoldsi* sp. nov. (Cnidaria, Scyphozoa, Coronatae, Atollidae): A New Species of Coronate Scyphozoan Found in the Eastern North Pacific Ocean †

**DOI:** 10.3390/ani12060742

**Published:** 2022-03-16

**Authors:** George I. Matsumoto, Lynne M. Christianson, Bruce H. Robison, Steven H. D. Haddock, Shannon B. Johnson

**Affiliations:** Monterey Bay Aquarium Research Institute, Moss Landing, CA 95039, USA; lynne@mbari.org (L.M.C.); robr@mbari.org (B.H.R.); haddock@mbari.org (S.H.D.H.); sjohnson@mbari.org (S.B.J.)

**Keywords:** *Atolla*, new species, Pacific, coronate, Scyphozoa, ROV, trawl

## Abstract

**Simple Summary:**

This paper describes an unusually large and distinctive deep-sea red medusa with coiled tentacles in the family Atollidae. This family is monogeneric with ten presently accepted species in the genus *Atolla*. The new medusa is molecularly and morphologically distinct from the five species that we have been able to sample and morphologically distinct from all ten previously described species. We have also observed and collected samples from another two potentially new species. The ocean provides over 98% of the available living space on our planet and we still do not know who is living there or how they interact with one another. This paper adds to the increasing number of new deep-sea species being described as we increase our exploration, and as advances in undersea technology and genetic sequencing become more available.

**Abstract:**

We have observed and collected unusual specimens of what we recognize as undescribed types of the genus *Atolla* over the past 15 years. Of these, there appear to be three potentially different types. One of these has now been genetically sequenced and compared both morphologically and molecularly with five other *Atolla* species that have been found in the eastern Pacific. This new variant is so morphologically distinct from other previously described *Atolla* species that we believe it can be described as a new species, *Atolla reynoldsi* sp. nov. This species along with two additional types may comprise a new genus. It is also clear that a more accurate and diagnostic morphological key for the genus *Atolla* needs to be developed. This paper will also provide some potential starting points for a new key to the genus.

## 1. Introduction

The subclass Coronamedusae Calder, 2009 [1] is within the class Scyphozoa Götte, 1887 [2] and contains one order (order Coronatae Vanhöffen, 1892 [3]). There are several families in the Coronatae including the family Atollidae Hickson, 1906 [4] which is monogeneric (*Atolla* Haeckel, 1880 [5]) with ten potential species [6], some of which are tentative. *Atolla* specimens have been found in every ocean basin in the world [7], but their contributions to the trophic ecology of pelagic ecosystems have been largely overlooked [8].

The well-documented species of *Atolla* are *A. vanhoeffeni*, *A. chuni*, and *A. gigantea*. Each of these have morphological characteristics that make them relatively easy to identify and are unique. Many of the other species can be separated into different taxa using the current taxonomic keys [8,9,10,11] but this paper shows that some of these morphological characteristics are not useful.

*Atolla verrillii* and *A. valdiviae* are both considered to be doubtful species [6] (perhaps both are *A. wyvillei*). *Atolla tenella* is described as having very distinctive pigmentation on the margin of the exumbrella but other than the illustration in the original description [12], we have not seen an image or mention of this pigmentation despite specific identifications in the literature [13], and Russell [11] considered the validity of the species as uncertain.

*Atolla chuni* was first described from two specimens collected south of the Cape of Good Hope [3] and is distinguished from other *Atolla* species by distinct papillae (‘pearls’) on the lappets. Larson reviewed additional descriptions of *A. chuni*, which he regarded as endemic to the Southern Ocean, and added observations from 1168 specimens [8].

There is still little known about the identity, behavior, or distribution of *Atolla*, one of the most common coronate scyphozoans in the deep ocean. In situ observations from crewed submersibles and remotely operated vehicles have revealed a number of observations on *Atolla*’s swimming and behavior. It is rare that a net tow or a trip to the deep does not reveal one or more *Atolla*. A four-year net study to examine one species (*A. wyvillei*) in the Bay of Biscay [14] found two distinct species (*A. wyvillei* and *A. parva*) with no apparent seasonal or depth differences. Hunt and Lindsay [15] discussed the potential for the hypertrophied tentacle that *Atolla* often exhibits for prey capture (also discussed in an unpublished report by Walker [16]). Direct observations using submersibles revealed that *Atolla* can capture prey including *Nanomia* (a phyosnect siphonophore) with this tentacle [17]. Additionally, Moore et al. [18] observed the large red caridean shrimp *Notostomus robustus* feeding on *A. wyvillei*—this feeding continued even after collection.

Thirty years of remotely operated vehicle observations with MBARI have revealed numerous observations of *Atolla* with trailing tentacles—so, when we find jellies that look like *Atolla* but are lacking the long trailing tentacle, it makes us stop and take a longer look. Over the past 15 years using a variety of ROVs, we have collected numerous specimens of three types of *Atolla*-like jellies that lack trailing tentacles. We have also collected other *Atolla* species and have found that existing species keys are often incomplete making it difficult to identify specimens to the species level [8,9,10,11]. Despite this, the new species described here is very distinctive and easy to differentiate from all other *Atolla* we have collected.

## 2. Materials and Methods

Specimens used in this study were collected using a diversity of means. Like many of the earlier scientific studies, some were collected using midwater trawls from the RV *Western Flyer* (Monterey Bay, Southern California, and the Gulf of California) as well as the RV *Kilo Moana* and the RV *Ka’imikai-O-Kanaloa* (In the Hawaiian Islands). The majority of the specimens were collected with the remotely operated vehicles ROV *Tiburon*, ROV *Ventana*, and ROV *Doc Ricketts* using the RV *Point Lobos*, RV *Rachel Carson*, and the RV *Western Flyer* in the Gulf of California, Southern California Bight, and Monterey Bay. Additional materials (*Atolla tenella*) were provided by Kevin Raskoff from the Arctic Ocean [13].

### 2.1. ROV Collections

We used three remotely operated vehicles (ROV *Ventana*, ROV *Tiburon*, and ROV *Doc Ricketts*) owned and operated by the Monterey Bay Aquarium Research Institute (MBARI) [19]. High-Definition video cameras were mounted on these vehicles and the video signal was conveyed to the surface support vessel specific for each ROV (ROV *Ventana*—R/V *Point Lobos* and R/V *Rachel Carson*; ROV *Tiburon* and ROV *Doc Ricketts*—R/V *Western Flyer*) through the ROV’s tether. At the surface, the video signal was viewed on a high-resolution monitor and was recorded on high-definition tape. More recent observations are with a 4K camera and digital recordings. Comments and descriptions of what is on the recordings are recorded on the audio track of the recording during the dive and can be accessed as needed by MBARI staff or collaborators. Additional environmental data (depth, location, temperature, dissolved oxygen, and salinity) during each dive are collected by instruments on the vehicle and the surface ship and integrated into an accessible and comprehensive relational database (http://dsg.mbari.org/dsg/home accessed on 7 March 2022) that is available to the public.

Specimens for this study were collected in 6.5 L ‘detritus’ samplers, designed for the gentle capture of delicate material in midwater [20], or in a ‘suction’ sampler consisting of a transparent funnel and two meters of flexible tubing leading back to 12 separate 6 L collection cylinders within the ROV tool sled. For the detritus samplers, the ROV pilot positioned the vehicle so that the open cylinder of the sampler enclosed the medusa, then the doors at either end were gently closed by hydraulic rams. For the suction sampler, the funnel, attached to a clear plexiglass tube and flexible tubing, could be extended in front of the ROV [20]. When a medusa was near the wide opening of the funnel, the suction pump was turned on and the animal was gently collected and deposited into a sample container, which was then replaced by an empty container.

Specimens were removed from the collection containers and photographed, if possible, prior to freezing portions or whole animals in liquid nitrogen for later molecular analysis. The remainder of each specimen was then preserved for morphological analysis. One specimen of the new species described in this paper was frozen, dried, and used for CHN elemental analysis as part of a different research project.

### 2.2. DNA Extraction and Amplification

Genomic DNA was isolated from frozen tissue samples using the Monarch Genomic DNA Purification Kit (New England Biolabs, Ipswich, MA, USA) or the DNeasy DNA Blood and Tissue kit (Qiagen, Germantown, MD, USA).

We amplified *18S* rDNA gene fragments (1793 bp) with the MitchA and MitchB primers [21]. We had limited success amplifying a few species with universal *COI* Folmer primers [22]; therefore, we designed new primers based on successful amplifications and on published sequences. Primer sequences were anchored in more conserved areas and analyzed with PrimerQuest program, IDT, Coralville, Iowa, USA (www.idtdna.com/SciTool last accessed 12 December 2018). The *COI* fragments (697 bp) of the newly-described *Atolla reynoldsi* sp. nov. were amplified using forward primer *Atolla*white_F2 (CGGGTCCAGTAATGGGAGAAG) and reverse primer *Atolla*GB_R2m1(TGAGCTCATACAACAAAACCAAG), and *Atolla* species B was amplified using forward primer *Atolla*white_F2 and reverse primer *Atolla*GB_R3(CATATGATGRGCYCATACWAYAAAYCCT). All other *Atolla* and coronate species were amplified with the primers *Atolla*GB_F2 (CTGGRCCTTTAATGGGTGATG) and *Atolla*GB_R2(TGAGCTCATACAACAAARCCT). All fragments were amplified with Phusion High-Fidelity PCR Master Mix with HF buffer (New England BioLabs, Ipswich, MA, USA) in a Veriti PCR thermal cycler (Life Technologies, Carlsbad, CA, USA). PCR conditions were: 98 °C for 30 s; 35 cycles of 98 °C for 30 s, 48 °C for 10 s, and 72 °C for 10 s; and a final extension of 72 °C for 5 min. Gene fragments were sequenced bi-directionally with PCR primers and the BigDyeTerminator v3.1 (Life Technologies, Carlsbad, CA, USA) sequencing kit and analyzed on a 3500xL Genetic Analyzer (Life Technologies, Carlsbad, CA, USA).

### 2.3. DNA Analyses

Bi-directional sequences were assembled and edited sequence fragments with Geneious Prime (v.2022.0.1, https://www.geneious.com last updated 13 January 2022). We aligned data with MUSCLE and estimated the best substitution model with AIC [23] with ModelTest within Geneious Prime. We included all available data from GenBank for closely related species, including *Periphylla periphylla*, *Paraphyllina* sp., *Periphyllopsis* sp., *Nausithoe* sp., *Atorella* sp., species of *Atolla*, and *Linuche* as an outgroup (accession numbers included in the phylogenies). Mitochondrial data were translated with the invertebrate mitochondrial genetic code to detect the presence of stop codons or pseudogenes. We estimated Bayesian phylogenies for *18S* rDNA and *COI* mtDNA separately with MrBayes (v.3.2.7a, [24,25]). Bayesian analyses included multiple runs that ranged from 5–10^8^ generations where we sampled and printed every 1000 generations with six chains after we discarded the first 10% of data. We also estimated likelihood trees with the program IQtree 2 [26,27] with 1000 bootstrap replicates. We trimmed alignments to exclude missing data for likelihood analyses. Phylogenies were visualized with FigTree (v.1.4.4, http://tree.bio.ed.ac.uk/software/figtree/ accessed on 13 January 2022). Sequences were analyzed using the GTR + I + Γ selection model.

## 3. Results

### 3.1. Collection Information

#### 3.1.1. *Atolla* Species Sequenced

**Figure 1:**
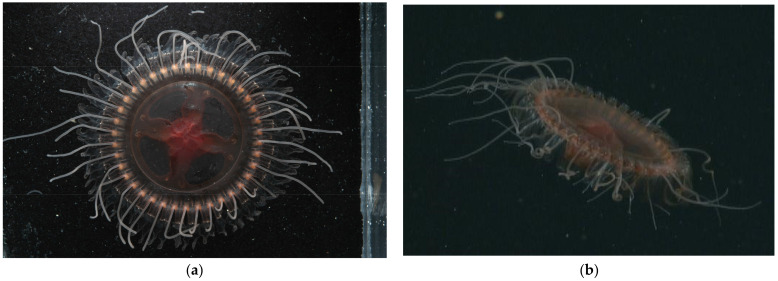
Images of *Atolla reynoldsi* sp. nov. from T960 on 4 April 2006. (**a**) Laboratory photo of *Atolla reynoldsi* sp. nov. (photo by Rob Sherlock). Diameter from margin to margin (excluding lappets) is 8.5 cm and tentacles were coiled in situ. (**b**) In situ image of *Atolla reynoldsi* sp. nov. The spikes and spike ridges on the lappets and the coiled tentacles are visible.

**Figure 2:**
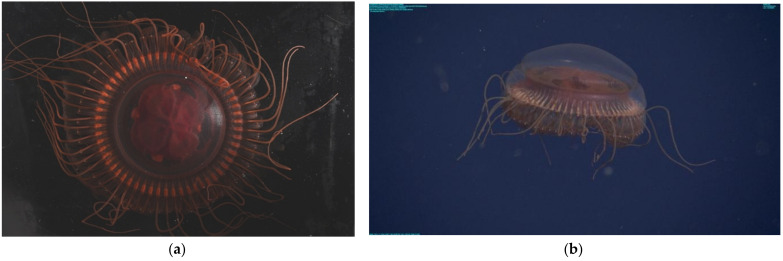
(**a**) Laboratory photo of *Atolla* species A taken in the lab (photo by SHDH) of the specimen collected on 30 October 2021 (D1399). Diameter from margin to margin (excluding lappets) is 8.5 cm. (**b**) In situ image of *Atolla* species A (D1402) photographed on 14 November 2021 at a depth of 1913 m, 5.4 cm in diameter.

**Figure 3:**
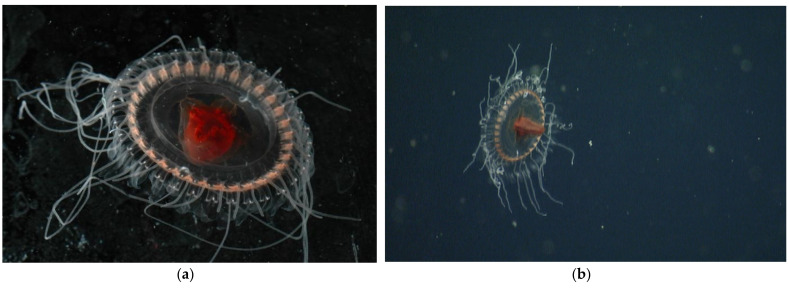
(**a**) Laboratory photo of *Atolla* species B (photo by Rob Sherlock) of the specimen collected on 14 April 2007 (T1088). (**b**) In situ image of *Atolla* species B (T1088) photographed on 14 April 2007 at a depth of 2570 m.

We sequenced 34 *Atolla* specimens that were collected between 2005 and 2021 (Table 1). Species identification was based on some existing keys [8,9,10,11] but there is not a key that includes all ten potentially valid species [6]. During the last few years, it became apparent to us that identifying *Atolla* to the species level was not straight forward and that some of the traits used in the keys were suspect. We also observed three types of *Atolla* that were clearly different from the described species (Table 2, Table 3 and Table 4 and Figure 1, Figure 2 and Figure 3). All three types lacked the characteristic *Atolla* hyperextended trailing tentacle and presented with a Greek-cross gut morphology that was different from that seen in the other described species (see Section 3.2.3). 

#### 3.1.2. *Atolla reynoldsi* sp. nov.

Table 2 and Figure 1 refer to *Atolla reynoldsi* sp. nov. *Atolla reynoldsi* sp. nov. has 26–39 tentacles that are coiled in situ, a Greek-cross gut morphology with smooth edges, spiked ridges and papillae on the rhopaliar pedalia and no trailing tentacle. 

#### 3.1.3. *Atolla* Species A

Table 3 and Figure 2 refer to *Atolla* species A. *Atolla* sp. A has 59–64 tentacles, a Greek-cross gut morphology with both invaginations and evaginations, no spiked ridges or papillae on the rhopaliar pedalia, and no trailing tentacle. 

#### 3.1.4. *Atolla* Species B

Table 4 and Figure 3 refer to *Atolla* species B. *Atolla* sp. B has 32–60 tentacles, a Greek-cross gut morphology with smooth edges, no spiked ridges, but may have some papillae on the rhopaliar pedalia, and no trailing tentacle. 

### 3.2. Morphological Distinctions

#### 3.2.1. Pigmentation

**Figure 4:**
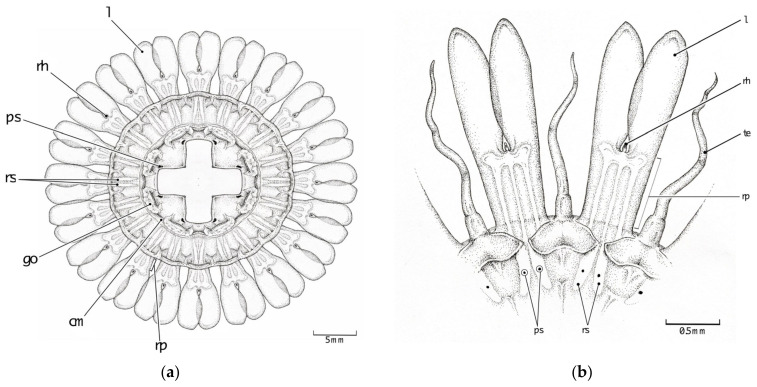
Line drawing showing pigmentation location and patterns for pigmentation observed on the (**a**) oral side of *Atolla vanhoeffeni* (modified from Russell 1957 [28]) and the (**b**) aboral side of *A. tenella* (modified from Hartlaub 1909 [12]). cm coronal muscle; go gonad; l lappet; rh rhopalium; te tentacle; rs radial septa; ps pigment spot; rp rhopaliar pedalia.

Currently, only two of the described *Atolla* species have pigment spots as one of their diagnostic characters, whereas the new species has none. *Atolla vanhoeffeni* has eight very distinct pigment spots that were first identified by Vanhoffen [28] and used by Russell [29] to erect the new species *A. vanhöffeni*. These pigmentation spots are on the subumbrellar walls of the stomach where the gastric cavity begins to narrow (Figure 4a); they are not pores. Hartlaub [12] described another new species (*A. tenella*) that has two pigment spots on the margin and centered on the rhopaliar pedalia (Figure 4b). While the pigmentation for *A. vanhoeffeni* can be easily found on specimens, we have not observed or seen any photographs of the pigmentation for *A. tenella*. The specimens used for the original species description were small (5–10 mm), and since our specimens came from the same expedition as Raskoff et al. [13] and these were identified as *A. tenella*, we have kept that identification (note, a preserved sample from that expedition did not have pigment spots) and have tentatively identified many of our Hawaiian samples as *A.* aff. *tenella*? based on a close molecular similarity with the Arctic Ocean *A. tenella* (Appendix A Table A3).

#### 3.2.2. Papillae

**Figure 5:**
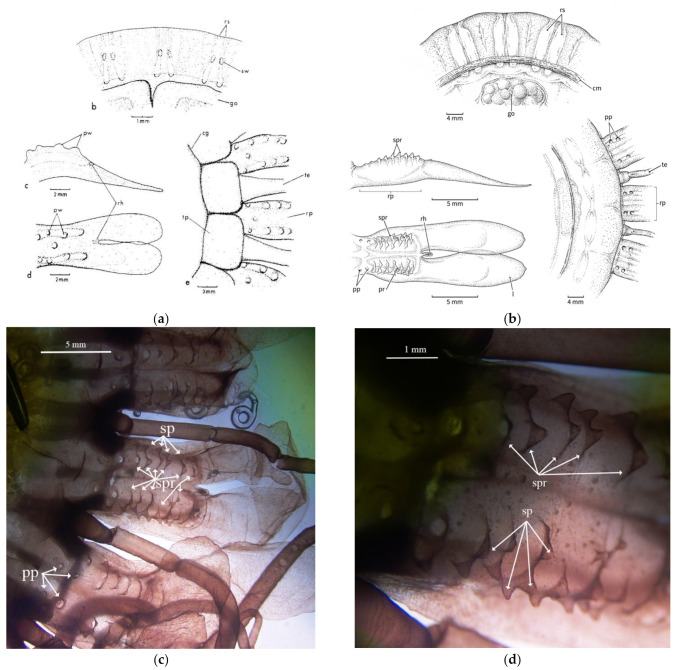
(**a**) Line drawing showing the pedaliar wart (pw) and septal wart (sw) pattern for *Atolla chuni* (from Larson [3] and used with permission under license number 5203151138336) and (**b**) the spikes on the rhopaliar pedalia for *Atolla reynoldsi* sp. nov. (drawn from photographs). Dissection microscope images (**c**) 7.5× and (**d**) 30× of the papillae (pp), spikes (sp), and the spike ridges (spr) of *Atolla reynoldsi* sp. nov (D1369D1). There were no septal warts observed. l lappet; rp rhopaliar pedalia; rh rhopalium; cm coronal muscle; te tentacle; go gonad; rs radial septa; pw pedaliar wart; sw septal wart; pp papillae; sp spike; spr spiked ridge.

*Atolla chuni* is the only described species of *Atolla* with protrusions labeled as warts on the exumbrellar surface [8]. *A. chuni* also has paired warts (sw) on top of the radial septa (Figure 5a) and another 7–9 warts (with one in the center and the others in two lateral rows) on the rhopaliar pedalia (Figure 5a). We are using the term papillae rather than warts for *A. reynoldsi* sp. nov. for the solitary protrusions as papillae is more commonly used in the literature for cnidarians. We are also using the term spikes to reflect the protrusions on ridges (for *Atolla reynoldsii* sp. nov.) as they have a variety of morphologies (Figure 5b–d) and are not simple rounded warts as in *A. chuni.* We have not included *A. chuni* in our analysis as we have never found a specimen that meets this description. *Atolla reynoldsi* sp. nov. has distinct ridges (~7) on each side of the rhopaliar pedalia that have spikes that are rounded close to the margin but pointed closer to the end of the pedalia. There are four solitary papillae closest to the body disc and no papillae over the radial septa. *Atolla* sp. A lacks papillae or spikes on ridges while *Atolla* sp. B has papillae (small and lined up in two rows like those of *A. chuni*) on the rhopalia but not over the septa. There are no spiked ridges in *Atolla* sp. B.

#### 3.2.3. Stomach Morphology

**Figure 6:**
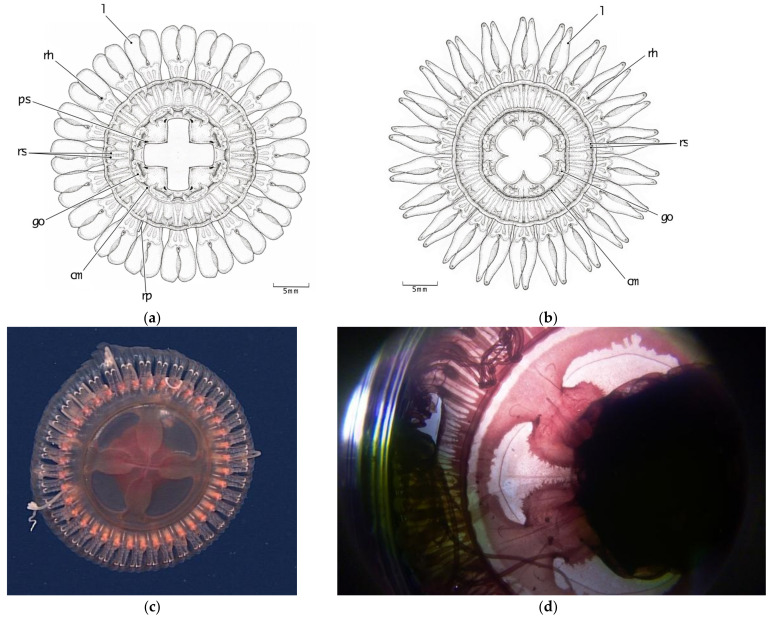
Line drawings showing the stomach patterns for (**a**) *Atolla vanhoeffeni*, (**b**) *Atolla wyvillei*, (**c**) *Atolla reynoldsi* sp. nov.—(D1369) in situ, 8.5 cm in diameter showing four narrow bases at the center of the medusa that expand into a vase-like shape before ending with a shallow indentation. (**d**) *Atolla* sp. A (D1399) in lab, 8.5 cm in diameter showing a much more rounded expansion with a much deeper indentation and the edges of the rounded expansion show invaginations near the center of the stomach and evaginations around the margin near the coronal muscle (cm). go gonad; l lappet; rh rhopalium; te tentacle; rs radial septa; ps pigment spot; rp rhopaliar pedalia.

There have been two basic stomach patterns (Figure 4b and Figure 6a) described for species within the genus *Atolla* [28]. *Atolla vanhoeffeni* presents a simple cross-shaped pattern (Figure 6a) while the basal stomach pattern for the other *Atolla* species is more similar to a four-leaf clover (Figure 6b). All three new *Atolla* types observed in Monterey are not only larger than the other described species (with the exception of *Atolla gigantea*), but also exhibit a different stomach pattern. We have termed this new morphology a Greek-cross shape (similar to Maltese cross shape with oval arms) and it presents as a thin base that then expands into a vase shape and ends with a shallow indentation near the edge of *Atolla reynoldsi* sp. nov. (Figure 6c) and *Atolla* sp. B, or a much more globular shape with very deep indentations near the margin edge for *Atolla* sp. A. (Figure 6d). Both *Atolla reynoldsi* sp. nov. and *Atolla* species B have this Greek-cross stomach pattern with smooth edges (Figure 6c) while *Atolla* species A has the Greek-cross stomach pattern with evaginations along the stomach and invaginations close to the center of the medusa and evaginations at the margin near the coronal muscle (Figure 6d). 

#### 3.2.4. Radial Septa

**Figure 7:**
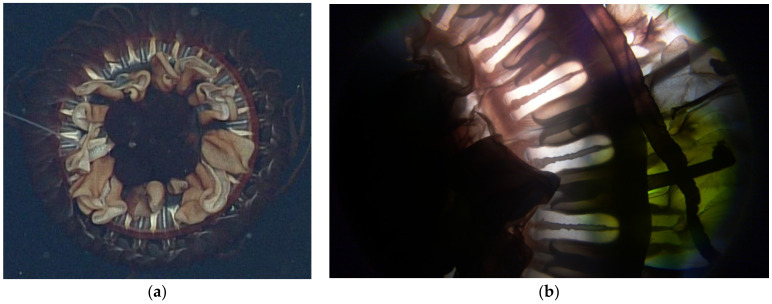
(**a**) In situ image of *Atolla gigantea* (D915) showing what appears to be divergent septa and (**b**) a photograph of the same specimen preserved in 5% formalin showing what appear to be straight septa. This specimen was 7.6 cm in diameter.

Radial septa are easily observed both in situ and in preserved specimens and have been used as a diagnostic character in keys for the genus *Atolla*. Our observations have revealed that preservation has an impact on the morphology of the septa. Specifically, for *A. gigantea*, the septa are clearly divergent when examining in situ frame grabs (Figure 7a) but appear to be straight when looking at preserved specimens (Figure 7b). This could be simply due to contraction of the bell, but it still makes the use of this morphology suspect for taxonomy as the amount of contraction would likely vary with fixative type and concentration.

#### 3.2.5. Tentacles

The appearance of a hypertrophied tentacle is generally used as a diagnostic character for the genus *Atolla*. The number of tentacles has been used as another diagnostic but there appears to be a great deal of variability in the number for each species. At this point, there does not seem to be enough confidence to use tentacle number as a diagnostic nor (if these three new species are to be kept in the genus *Atolla*) can the hypertrophied tentacle be used. Nine of the ten specimens of *Atolla reynoldsi* sp. nov. observed had coiled tentacles; the tenth was able to coil some of the tentacles but then released the coil to display tentacles more similar to other *Atolla* species.

### 3.3. Molecular Results

We sequenced three individuals of *A. reynoldsi* sp. nov. for the *COI* mtDNA and *18S* rDNA fragments in addition to 30 new sequences of close relatives for statistical analyses. (Table 1, GenBank accession #’s OM214492-OM214523 and OM260056-ON260088). We sequenced nine other coronate species for *18s* rDNA and one for *CO1* mtDNA (Table 5, GenBank accession numbers OM201135-OM201143 and OM237455).

**Figure 8:**
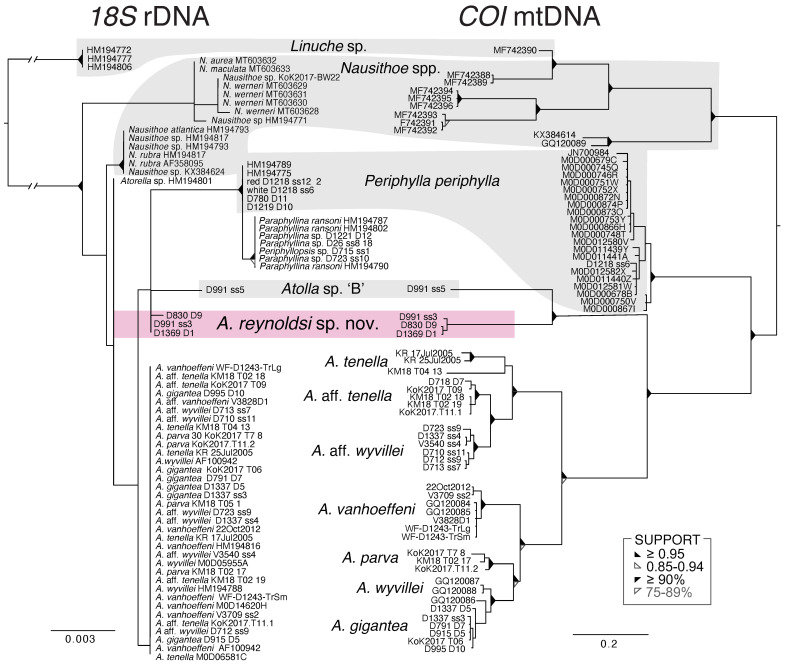
Bayesian and Likelihood estimates of phylogenetic trees for coronate jellies with an 1826 base pairs (bp) alignment of the *18S* rDNA fragment with a GTR + I + Γ selection model and an 892 bp alignment of the *COI* mtDNA fragment with a GTR + Ι + Γ selection model. Posterior probabilities and bootstrap results displayed as triangles (see legend). Support of nodes with below threshold value not shown.

Sequencing efforts for *Atolla* sp. A are ongoing: we have gotten some preliminary *18S* rDNA sequences but they are not included in Figure 8 as they are only ~300 bp long and identical to the sequence obtained for *Atolla* sp. B. The alignments of *18S* rDNA were conserved among *Atolla*, *Periphylla*, *Periphylopsis*, *Linuche*, and *Nausithoe* species and resulted in very few mutations. As a result, phylogenies were mostly unresolved, especially within genera. However, *Atolla reynoldsi* sp. nov. was distinct from all other *Atolla* species (Figure 8).

The *COI* mtDNA alignments were more informative and provided delineation among and even within species from distinct localities with full Bayesian and likelihood support (Figure 8). *Atolla reynoldsi* sp. nov. differed from its closest relative, another undescribed type of *Atolla* sp. ‘B’ by about ~22% for the GTR + I + Γ selection model. This differentiation was far greater than among many other described species of *Atolla* (Figure 8). We do not yet have *COI* mt DNA for *Atolla* sp. A.

## 4. Discussion

### 4.1. Systematics

Class Scyphozoa Götte, 1887 [1]

Subclass Coronamedusae Calder, 2009 [2]

Order Coronatae Vanhöffen, 1892 [3]

Family Atollidae Hickson, 1906 [4]

Genus *Atolla* Haeckel, 1880 [5]

*Atolla reynoldsi* sp. nov.

Figure 1, Figure 5b–d and Figure 6c.

Diagnosis: *Atolla reynoldsi* sp. nov. can have from 26–39 tentacles and rhopalia. The overall shape is flattened although the center zone is a rounded dome, albeit not very tall (Figure 1). The tentacles in situ are usually coiled and a hypertrophied tentacle has not been observed. There are ~nine lateral ridges along the pedalia that have some spikes of various heights (Figure 5b–d). The gut has a distinctive Greek-cross morphology (Figure 6c). Diagnostic characters separating this new species from extant *Atolla* species include the spiked ridges and papillae on the exumbrellar surface of the rhopaliar pedalia, the ability to coil the tentacles, the Greek-cross gut morphology, and the lack of a hyptertrophied tentacle. The gonads are oval when immature but become large and horseshoe-shaped when mature. The radial septa are straight or slightly divergent and extend beyond the coronal muscle.

Type material: The type specimen was collected on 30 June 2021 at 3189 m depth, at 35°29′58.0776″ N and 123°59′55.536″ W in Monterey Bay, California. The holotype specimen and three paratype specimens have been deposited at the California Academy of Sciences (Holotype: CASIZ no. 233651; Paratypes: CASIZ 233650, CASIZ 233652, and CASIZ 233653). Two additional paratypes are housed at the Monterey Bay Aquarium Research Institute (MBARI). A total of ten specimens have been collected between April 2006 and June 2021 (Table 1) in Monterey Bay (eastern North Pacific Ocean) at depths between 1013 and 3189 m.

Etymology: Named after the first volunteer at the Monterey Bay Aquarium (Jeff Reynolds) who guarded a beached whale on Del Monte Beach overnight so that the Aquarium could retrieve it and prepare it for eventual overhead display.

Systematic remarks: The order Coronatae is identified by the separation of the exumbrella into two concentric zones by a circular coronal groove. The central zone is a circular disc or dome while the marginal zone is divided by radiating grooves into thickened pedalia, with peripheral lappets. The presence of more than eight rhopalia place it into the family Atollidae, which is currently monogeneric.

The previously described number of rhopalia in the genus *Atolla* is 16–32. However, *Atolla reynoldsi* sp. nov. has up to 39 rhopalia and the *Atolla* sp. A and *Atolla* sp. B have up to 64 rhopalia. While it is possible that these types with 32 or more rhopalia might be a new genus, we are not comfortable at this time in making this recommendation as we have not examined all 10 putative species or completed the molecular analysis for *Atolla* sp. A and *Atolla* sp. B. Therefore, we recommend that the new diagnosis for the family Atollidae be modified to include up to 64 tentacles and rhopalia. We are in the process of writing up new species descriptions for *Atolla* sp. A and *Atolla* sp. B as soon as we complete our molecular analysis of these two types. 

### 4.2. Molecular Analysis

The *18S* rDNA fragment was highly conserved and resulting phylogenies were paraphyletic among *Atolla*, *Periphylla periphylla*, *Paraphyllina*, and *Periphyllopsis* species. Despite unresolved polytomies, the *18S* fragment clearly delineated between *Atolla reynoldsi* sp. nov. and *Atolla* sp. B, while other species of *Atolla* had identical residues.

The *COI* mtDNA locus provided better resolution and stronger support for the delineation of species and for the inclusion of *A. reynoldsi* sp. nov. into the *Atolla* genus. *Atolla reynoldsi* sp. nov. differed from its closest relatives, two undescribed species of *Atolla* (sp. A and B) by about ~22% for the GTR + I + I selection model. This differentiation was far greater than many other described species of *Atolla* but they were still closer to *Atolla* than other genera in the order (Figure 8). *Atolla reynoldsi* sp. nov. and *Atolla* spps. A and B were more distantly related to other *Atolla* species, although there was full likelihood and Bayesian support for their inclusion into the *Atolla* genus.

The remainder of *Atolla* species were more closely related and their interrelationships were less clearly resolved. *Atolla tenella* from the Arctic region (as identified by Raskoff et al. [13] and what we identified as *A.* aff. *tenella* from Hawaii (based on having 30 tentacles) were distinct from each other, and neither had the pigment spots that were indicative of the species in the original description [12]. Published *COI* sequences for *A. wyvillei* from the North Atlantic also differed from the *COI* fragment of *A.* aff. *wyvillei* from the Gulf of California and Southern California. *Atolla gigantea*, *A. vanhoeffeni*, and *A. parva* were more easily identified morphologically and sequence data were congruent with morphology.

## 5. Conclusions

Our investigations reveal that there are types of *Atolla*-like coronates that do not fall within the current taxonomic descriptions of the family Atollidae or the genus *Atolla*. Until more information can be gathered, we are proposing that *Atolla reynoldsi* sp. nov. remain within *Atolla* (along with the other two potential new types A and B) but that more work needs to be completed to clarify their placement within the coronates. We do plan on continuing this work and describing these two new types. 

Despite the lack of an adequate key, it is clear that that *Atolla reynoldsi* sp. nov. is molecularly distinct from the *Atolla* species that we have been able to collect and that it is morphologically distinct from all ten described *Atolla* species (although sharing the presence of papillae with *A. chuni*).

The two additional types (*Atolla* species A and *Atolla* species B) may likewise be new species but we do not have enough samples at this time to make that claim. All three types (*Atolla reynoldsi* sp. nov., *Atolla* species A, and *Atolla* species B) may need to be placed into a new genus due to their distinct stomach morphology and the lack of a trailing tentacle, but until further work is completed, we recommend that they remain within the genus *Atolla* and that the family description (Atollidae) be modified to include 16–62 rhopalia rather than 16–32 rhopalia.

Current keys for *Atolla* species need to be revised as there are no keys that include all ten species, the number of tentacles is more variable than original authors had noted, and the use of radial septa in the keys is problematic, as determining if they are divergent or straight is somewhat subjective and can be changed by preservation. We recommend that additional examination of all ten described species be completed, ideally with specimens from the type localities, and that a more accurate key be developed for the described species. A table listing diagnostic traits for species included in this analysis is provided as an appendix (Table 2).

Erection of a better dichotomous key for the genus will require better identification of the putative existing species so we suggest that specimens from the original locations be obtained and photographed/sequenced in order to create a more accurate key. It is possible that some of the described species are not valid species and will need to be placed into an existing species. *Atolla vanhoeffeni* can be clearly distinguished morphologically from all other extant species (based on the pigment spots) and it also groups as a separate species molecularly. While *A. tenella* may also have pigmentation spots, we have been unable to find any photographic evidence and the validity of this species should be examined as it might just be *A. wyvillei* [11]. The use of radial septa orientation (divergent or straight) is problematic as fixation causes this to change. *Atolla wyvillei* is supposed to have septa that pass the coronal muscle but we have found that other specimens that classify as *A. reynoldsi* sp. nov., *A. parva*, and *A gigantea* also have septa that extend beyond the coronal muscle. *Atolla chuni* is known to have papillae or warts and these have been well documented by Larson and are quite different from those of *A. reynoldsi* sp. nov., but we were not able to find *A. chuni* specimens to sequence.

## Figures and Tables

**Table 1:** Physical measurements and accession numbers for the collected *Atolla* specimens used for sequencing *COI* and 18S rDNA.

Species ID	Date	Accession Number *18s*	Accession Number *COI*	Sample ID	Depth m	Lat Decimal	Long Decimal	Tentacle Number
*A. gigantea*	10 August 2015	OM260068	OM214504	D0791D7	1112	36.531771	−122.507713	24
*A. gigantea*	14 December 2016	OM260069	OM214505	D0915D5	1016	36.260438	−122.593946	24
*A. gigantea*	10 December 2017	OM260070	OM214503	D0995D10	897	36.748584	−122.103112	24
*A. gigantea*	19 March 2017	OM260073	OM214506	KOK-T06	1584–888	19.275	156.133333	59
*A. gigantea*	30 May 2021	OM260072	OM214502	D1337ss3	1253	33.850068	−119.850222	24
*A. gigantea*	30 May 2021	OM260071	OM214501	D1337D5	936	33.850169	−119.651757	24
*A. parva*	20 March 2017	OM260077	OM214507	KOK-T7/8	838–581	19.483333	156.13333	30
*A. parva*	21 March 2017	OM260076	OM214509	KOK17-T11	1700–1115	19.666667	156.13333	30
*A. parva*	6 November 2018	OM260074	OM214508	KM-T02.17	NA	19.426389	156.408611	26
*A. parva*	7 November 2018	OM260075	NA	KM18-T05	NA	19.318333	156.185833	26
*A. tenella*	17 July 2005	OM260079	OM214511	KR−17	NA	Arctic		NA
*A. tenella*	25 July 2005	OM260080	OM214512	KR−25	NA	Arctic		NA
*A.* aff. *tenella*	2 March 2015	NA	OM214513	D718D7	1696	24.412664	−109.095732	22
*A.* aff. *tenella*	20 March 2017	OM260060	OM214515	KOK17-T09	1719–1033	19.483333	156.13333	24
*A.* aff. *tenella*	21 March 2017	OM260061	OM214516	KOK17-T11	1700–1115	19.666667	156.13333	30
*A.* aff. *tenella*	6 November 2018	OM260058	OM214517	KM-T02.18	NA	19.426389	156.408611	30
*A.* aff. *tenella*	6 November 2018	OM260059	OM214514	KM-T02.19	NA	19.426389	156.408611	30
*A. tenella*	7 November 2018	OM260078	OM214510	KM18 T04-13	2500	19.318333	156.185833	30
*A. vanhoeffeni*	22 October 2012	OM260084	OM214496	WF trawl	NA	36.699558	−122.049488	NA
*A. vanhoeffeni*	24 April 2013	OM260085	OM214497	V3709ss2	512	36.700040	−122.048422	NA
*A. vanhoeffeni*	27 May 2015	OM260081	OM214498	V3828D1	424	36.703304	−122.052176	NA
*A. vanhoeffeni*	31 January 2020	OM260082	OM214499	WF-trawl	NA	36.16666	−119.25	NA
*A. vanhoeffeni*	31 January 2020	OM260083	OM214500	WF-trawl	NA	36.16666	−119.25	NA
*A.* aff. *wyvillei*	18 March 2010	OM260067	OM214523	V3540ss4	626	36.705400	−122.053820	NA
*A.* aff. *wyvillei*	22 February 2015	OM260062	OM214518	D710ss11	746	24.277515	−109.360873	22
*A.* aff. *wyvillei*	24 February 2015	OM260063	OM214519	D712ss9	706	25.430911	−109.835949	22
*A.* aff. *wyvillei*	25 February 2015	OM260064	OM214520	D713ss7	774	25.446143	−109.848168	22
*A.* aff. *wyvillei*	9 March 2015	OM260065	OM214521	D723ss9	697	25.442727	−109.852024	22
*A.* aff. *wyvillei*	30 May 2021	OM260066	OM214522	D1337ss4	985	33.850169	−119.651757	24
*A reynoldsi* sp. nov.	5 December 2015	OM260086	OM214493	D0830D9	1013	36.688186	−122.118768	32
*A reynoldsi* sp. nov.	6 December 2017	OM260087	OM214492	D0991ss3	1878	36.548736	−122.541753	32
*A reynoldsi* sp. nov.	30 July 2021	OM260088	OM214494	D1369D1	3189	35.499466	−123.99876	38
*Atolla* type B	6 December 2017	OM260056	OM214495	D991ss5	1783	36.548400	−122.542593	60
*Atolla* type A	30 October 2021	OM260057	NA	D1399ss3	1253	36.700923	−122.067752	59

Temperature, salinity, and oxygen data are available in the appendix Table 1. Sample ID refers to the remotely operated vehicle and the dive number (V for ROV *Ventana*, T for ROV *Tiburon*, or D for ROV *Doc Ricketts*), trawls (T) aboard the RV *Kilo Moana* (KM), the RV *Ka’imikai-O-Kanaloa* (KOK), or the RV *Western Flyer* (WF). Two specimens of *Atolla tenella* collected in the Arctic (July 2005) were provided by Kevin Raskoff (KR). NA refers to information that is not available.

**Table 2:** Physical measurements and water parameters for the collected specimens of *Atolla reynoldsi* sp. nov.

Date	Sample ID	Depth m	Temp °C	Sal PSU	Oxy ml/L	Lat Decimal	Long Decimal	Tentacle Number	Diameter cm	Status
4 April 2006	T0960ss6	2848	1.657	34.621	2.29	36.573417	−122.5221505	38	13	CHN
7 April 2006	T0964ss5	1400	2.897	34.5	0.81	36.328862	−122.898496	26	NA	Frozen @
20 June 2006	T0996D6 damaged	1435	2.987	34.489	0.71	36.551573	−122.5109275	30	7.5	Damaged
20 October 2009	D0087D8 CASIZ 233652	1133	3.427	34.401	0.639	36.334888	−122.917099	32	8	Paratype @
9 November 2013	D0546D12 CASIZ 233653	2705	1.697	34.656	2.354	36.535624	−122.508432	39	7.3	Paratype @
3 August 2014	D0642D11	1500	2.777	34.541	0.858	36.533088	−122.509816	36	8	MBARI
5 December 2015	D0830D9	1013	3.861	34.436	0.348	36.688186	−122.118768	32	NA	Frozen $
6 December 2017	D0991ss3	1878	2.229	34.602	1.373	36.548736	−122.541753	32	5.8	MBARI $
10 August 2018	D1050D11 CASIZ 233650	1445	2.906	34.528	0.821	36.534146	−122.565410	26	7.7	Paratype
30 July 2021	D1369D1 CASIZ 233651	3189	1.576	34.665	2.470	35.499466	−123.99876	38	8.5	Holotype $

All specimens displayed coiled tentacles, no trailing tentacle, a Greek-cross gut morphology, and ridges with spikes on the rhopaliar pedalia. Sample ID refers to the remotely operated vehicle and the dive number (T for ROV *Tiburon*, or D for ROV *Doc Ricketts*). NA refers to information that is not available. Diameter is bell diameter from margin to margin excluding the lappets. The last specimen collected (30 July 2021) is the holotype at California Academy of Sciences (CASIZ 233651); this one was also sequenced ($). There are three paratypes at CASIZ (CASIZ 233650, CASIZ 233652, and CASIZ 233653), two paratypes at MBARI, and two specimens were frozen and sequenced ($). Specimen collected on 4 April 2006 was frozen for CHN elemental analysis as part of a different research project. Specimen collected on 20 June 2006 was damaged in transit to CASIZ and discarded. Short video clips are available (@) online at https://www.mbari.org/supplemental-for-matsumoto-etal-atolla-reynoldsi-new-species-pub/ (last accessed on 7 March 2022) and in the Supplementary Materials. Video S1: *Atolla reynoldsi* sp. nov. D0087, 20 October 2009; Video S2: *Atolla reynoldsi* sp. nov. D0546, 9 November 2013 and Video S3: *Atolla reynoldsi* sp. nov. T964, 7 April 2006.

**Table 3:** Physical measurements and water parameters for the collected specimens of *Atolla* species A.

Date	Sample ID	Depth m	Temp °C	Sal PSU	Oxy mL/L	Lat Decimal	Long Decimal	Tentacle Number	Diameter cm	Status
13 June 2002	T0439	1197	3.371	34.342	0.62	36.329753	−122.900502	59	NA	NA@
30 October 2021	D1399ss3	1253	3.264	34.515	0.764	36.700923	−122.067752	59	8.5	&
14 November 2021	D1402ss9	1913	2.210	34.603	1.434	36.543934	−122.536996	64	5.4	&@

These specimens displayed straight tentacles, no trailing tentacle, an evaginated Greek-cross gut morphology (see Section 3.2.3, and no papillae or spike ridges on the rhopaliar pedalia. The overall shape is tall with a distinctive large rounded dome. Sample ID refers to the remotely operated vehicle and the dive number (T for ROV *Tiburon*, or D for ROV *Doc Ricketts*). Diameter is bell diameter from margin to margin but excluding the lappets. Samples collected in 2021 are at MBARI (&), the specimen observed in 2002 was not collected. Short video clips are available (@) in the Supplementary Materials section and online at https://www.mbari.org/supplemental-for-matsumoto-etal-atolla-reynoldsi-new-species-pub/ (last accessed on 7 March 2022) Video S4: *Atolla gigantea* D0315, November 5 2011; Video S5: *Atolla* species A D1399, 30 October 2021 and Video S6: *Atolla* species A D1402 14 November 2021. NA refers to information that is not available.

**Table 4:** Physical measurements and water parameters for the collected specimens of *Atolla* species B.

Date	Sample ID	Depth m	Temp °C	Sal PSU	Oxy mL/L	Lat Decimal	Long Decimal	Tentacle Number	Diameter cm	Status
18 November 2004	T764	3247	1.603	34.6	2.61	36.329555	−122.899157	39	5.5	&
22 June 2006	T0998D4	3275	1.632	34.589	2.41	36.341234	−122.916458	32	1.7	&
14 April 2007	T1088D4	2570	1.807	34.437	2.12	36.551916	−122.502087	32	NA	NA
20 May 2014	D0613ss8	3302	1.697	34.652	2.47	36.501258	−122.866931	42	7.4	&
6 December 2017	D0991ss5	1783	2.363	34.592	1.271	36.548400	−122.542593	60	NA	$@

All specimens displayed coiled tentacles, no trailing tentacle, a Greek-cross gut morphology, and papillae but no spiked ridges. D0991sss5 was frozen and used for DNA sequencing ($) while the specimens from 2004, 2006, and 2014 are at MBARI (&). The specimen collected in 2007 was photographed in the lab but not examined further. Short video clips are available (@) in the Supplementary Materials section and online at https://www.mbari.org/supplemental-for-matsumoto-etal-atolla-reynoldsi-new-species-pub/ (last accessed on 7 March 2022) Video S7: *Atolla* species B D991 December 6 2017 and Video S8: *Atolla* species B D613 20 May 2014. NA refers to information that is not available.

**Table 5:** Coronate genera sequenced for rooting in the *18S* rDNA molecular tree.

Species ID	Date	Accession Number *18s*	Sample ID	Depth m	Lat Decimal	Long Decimal
*Nausithoe* sp.	23 March 2017	OM237455	KOK2017-BW22	30	20.756111	−157.255833
*Paraphyllina* sp.	27 May 2019	OM201136	D0026 ss8	2385	36.116665	−122.75
*Paraphyllina* sp.	9 March 2015	OM201137	D0723 ss10	651	25.442516	−109.852324
*Paraphyllina* sp.	18 November 2019	OM201138	D1221 D12	2088	36.545798	−122.538197
*Periphyllopsis* sp.	27 February 2015	OM201143	D0715 ss1	1761	28.182585	−119.599956
*Periphylla periphylla*	1 July 2015	OM201139	D0780 D11	534	36.15082	−124.2852
*Periphylla periphylla*	15 November 2015	OM201141	D1218 ss12	384	36.695557	−122.004649
*Periphylla periphylla*	15 November 2015	OM201140	D1218 ss6	392	36.698180	−122.010072
*Periphylla periphylla*	16 November 2015	OM201142	D1219 D10	923	36.544387	−122.537005

Temperature, salinity, and oxygen data are available in the appendix. Sample ID refers to the remotely operated vehicle and the dive number (D for ROV *Doc Ricketts*) followed by the type of collection (ss for Suction Sample and D for Detritus Sample).

## Data Availability

*COI* and *18S* sequence fragments have been deposited in GenBank (2022) with accession numbers OM260056-OM260088, OM214492-OM214523, OM201135-OM201143, OM237455, and OM202513.

## Supplementary Materials

The following videos are available online at https://www.mdpi.com/article/10.3390/ani12060742/s1, Video S1: *Atolla reynoldsi* sp. nov. D0087, 20 October 2009. Video S2: *Atolla reynoldsi* sp. nov. D0546, 9 November 2013. Video S3: *Atolla reynoldsi* sp. nov. T964, 7 April 2006. Video S4: *Atolla gigantea* D0315, 11/5/2011. Video S5: *Atolla* species A D1399, 30 October 2021. Video S6: *Atolla* species A D1402 14 November 2021. Video S7: *Atolla* species B D991 6 December 2017. Video S8: *Atolla* species B D613 20 May 2014.

## Appendix A

**Table A1:** Water parameters and collection information for sequenced *Atolla* and other coronate specimens. Sample ID refers to the remotely operated vehicle and the dive number (V for ROV *Ventana*, T for ROV *Tiburon*, or D for ROV *Doc Ricketts*), blue-water SCUBA dive (BW) or trawls (T) aboard the RV *Kilo Moana* (KM), the RV *Ka’imikai-O-Kanaloa* (KOK), or the RV *Western Flyer* (WF). Two specimens of *Atolla tenella* collected in the Arctic were provided by Kevin Raskoff (KR). Some information was not available (NA).

Species ID	Date	Sample ID	Depth m	Temp °C	Sal PSU	Oxy ml/L	Lat Decimal	Long Decimal
*A. gigantea*	10 August 2015	D0791D5	1112	3.809	34.457	0.373	36.531771	−122.507713
*A. gigantea*	14 December 2016	D0915D5	1016	3.852	34.467	0.411	36.260438	−122.593946
*A. gigantea*	10 December 2017	D0995D10	897	4.199	34.427	0.297	36.748584	−122.103112
*A. gigantea*	19 March 2017	KOK- T06	NA	NA	NA	NA	19.275	156.133333
*A. gigantea*	30 May 2021	D1337sss3	1253	4.278	34.464	0.332	33.850068	−119.850222
*A. gigantea*	30 May 2021	D1337D5	936	4.436	34.45	0.321	33.850169	−119.651757
*A. parva*	20 March 2017	KOK-T7/8	NA	NA	NA	NA	19.483333	156.13333
*A.* *parva*	21 March 2017	KOK17-T11	NA	NA	NA	NA	19.666667	156.13333
*A. parva*	6 November 2018	KM-T02.17	NA	NA	NA	NA	19.426389	156.408611
*A. parva*	7 November 2018	KM18 T05	NA	NA	NA	NA	19.318333	156.185833
*A. tenella*	17 July 2005	KR-17	NA	NA	NA	NA	Arctic	
*A. tenella*	25 July 2005	KR-25	NA	NA	NA	NA	Arctic	
*A.* aff. *tenella*	2 March 2015	D718D7	1696	2.633	34.615	1.212	24.412664	−109.095732
*A.* aff. *tenella*	20 March 2017	KOK17-T09	NA	NA	NA	NA	19.483333	156.13333
*A.* aff. *tenella*	21 March 2017	KOK17-T11	NA	NA	NA	NA	19.666667	156.13333
*A.* aff. *tenella*	6 November 2018	KM- T02.18	NA	NA	NA	NA	19.426389	156.408611
*A.* aff. *tenella*	6 November 2018	KM-T02.19	NA	NA	NA	NA	19.426389	156.408611
*A. tenella*	7 November 2018	KM18 T04-13	2500	NA	NA	NA	19.318333	156.185833
*A. vanhoeffeni*	22 October 2012	WF trawl	NA	NA	NA	NA	36.699558	−122.049488
*A. vanhoeffeni*	24 April 2013	V3709ss2	512	6.002	34.251	0.343	36.700040	−122.048422
*A. vanhoeffeni*	27 May 2015	V3828D1	424	6.876	34.179	0.924	36.703304	−122.052176
*A. vanhoeffeni*	31 January 2020	WF-D1243trawl	NA	NA	NA	NA	36.16666	−119.25
*A. vanhoeffeni*	31 January 2020	WF-D1243trawl	NA	NA	NA	NA	36.16666	−119.25
*A.* aff. *wyvillei*	18 March 2010	V3540ss4	626	5.077	34.319	0.195	36.705400	−122.053820
*A.* aff. *wyvillei*	22 February 2015	D710ss11	746	6.020	34.509	0.023	24.277515	−109.360873
*A.* aff. *wyvillei*	24 February 2015	D712ss9	706	5.997	34.516	0.032	25.430911	−109.835949
*A.* aff. *wyvillei*	25 February 2015	D713ss7	774	6.002	34.516	0.032	25.446143	−109.848168
*A.* aff. *wyvillei*	9 March 2015	D723ss9	697	5.956	34.516	0.034	25.442727	−109.852024
*A.* aff. *wyvillei*	30 May 2021	D1337ss4	985	4.436	34.45	0.321	33.850169	−119.651757
*A reynoldsi* sp. nov.	5 December 2015	D0830D9	1013	3.861	34.436	0.348	36.688186	−122.118768
*A reynoldsi* sp. nov.	6 December 2017	D0991ss3	1878	2.229	34.602	1.373	36.548736	−122.541753
*A reynoldsi* sp. nov.	30 July 2021	D1369D1	3189	1.576	34.665	2.470	35.499466	−123.99876
*Atolla* type A	30 October 2021	D1399-ss3	1253	3.264	34.515	0.764	36.7009226	−122.067752
*Atolla* type B	6 December 2017	D991ss5	1783	2.363	34.592	1.271	36.548400	−122.542593
*Nausithoe* sp.	23 March 2017	KOK2017-BW22	30	NA	NA	NA	20.756111	−157.255833
*Paraphyllina* sp.	27 May 2019	D0026 ss8	2385	1.858	34.592	1.943	36.116665	−122.75
*Paraphyllina* sp.	9 March 2015	D0723 ss10	651	6.199	34.512	0.025	25.442516	−109.852324
*Paraphyllina* sp.	18 November 2019	D1221 D12	2088	2.006	34.815	1.851	36.545798	−122.538197
*Periphyllopsis* sp.	27 February 2015	D0715 ss1	1761	2.839	34.819	1.087	28.182585	−119.599956
*Periphylla periphylla*	1 July 2015	D0780 D11	534	5.231	34.169	0.433	36.15082	−124.2852
*Periphylla periphylla*	15 November 2015	D1218 ss12	384	7.542	34.158	0.996	36.695557	−122.004649
*Periphylla periphylla*	15 November 2015	D1218 ss6	392	6.940	34.184	0.763	36.698180	−122.010072
*Periphylla periphylla*	16 November 2015	D1219 D10	923	4.237	34.416	0.321	36.544387	−122.537005

**Table A2:** Potential diagnostic trait table for species identification of *Atolla* based on existing keys [8,9,10,11] as well as original descriptions and our observations. Potential valid diagnostic traits are highlighted in yellow and we have grouped *A. tenella* with *A*. aff. *tennella* and *A*. *wyvillei* with *A.* aff. *wyvillei* as the molecular analysis shows that they are similar (Appendix A Table A3). Septa shape (divergent or straight), degree of extension into coronal muscle, and gonad shape are not considered by us to be valid diagnostic traits.

Species	*A. chuni* *	*A. parva*	*A. vanhoeffeni*	*A. tenella* and *A.* aff. *tenella*	*A. gigantea*	*A. wyvillei and A.* aff. *wyvillei*	*A. reynoldsi* sp. nov.	*Atolla* species A—tall, rounded dome	*Atolla* species B—White, very flat
Pigment spots	No	No	Yes 2 per quadrant	Yes? 2 per tentacle ^a^	No	No	No	No	no
Papillae	Yes	No	No	No	No	No	Yes	No	Yes, varies ^b^
Ridges with spikes on rhopalia	No	No	No	No	No	No	Yes	No	No
Stomach	Clover shaped	Clover shaped	Cross shaped	Clover shaped	Clover shaped	Clover shaped	Greek-cross shaped	Greek-cross shaped—evaginated	Greek-cross shaped
Tentacles	24	18–24	18–20	22–30	24, 28	22–30	26–39 coiled	59–64	32–60
Trailing tentacle	Yes	Yes	Yes	Yes	Yes	Yes	No	No	No
Septa path ^c^	?	Straight or slightly divergent, club shaped	Straight	Divergent in description; straight in our specimens	Divergent (preserved look straight)	Divergent	Straight or slightly divergent	Straight or slightly divergent	Straight
Septa extend to muscle ^d^	?	Yes	No	No	Yes	Yes ^e^	Yes	Yes	Yes
Gonad shape ^f^	Oval/bean	oval	Horseshoe	Circular with irregular edges	Horseshoe	Oval to large auricular	Oval but horseshoe shaped when mature	Horseshoe	Immature, horseshoe

Footnotes: * not observed or collected in this study; ^a^. Pigment spots not observed in our specimen or photos of *A. tenella*; ^b^. Papillae in *Atolla* sp. B vary from a few solitary papilla to two rows of papillae on the rhopalia, loss of papillae may be a result of fixation; ^c^. Path of the septa is not a good trait, as it is somewhat arbitrary and changes with preservation; ^d^. Septa extending into coronal muscle is also not a robust trait; ^e^. Septa not extending into coronal muscle is supposed to be diagnostic for *A. wyvillei*, but they do extend into the muscle; ^f^. Gonads change shape as they mature, going from a C-shaped outline to oval and then to fully folded horseshoe. Other species in the literature: *A.* *russelli*—might have a Greek-cross shaped gut (Lindsay et al. 2004 [30]) (16–22 tentacles divergent septa); 22 tentacles in original description; *A.* *bairdii*—Smithsonian holotype photo online, looks like *A gigantea* (22 tentacles, divergent septa); *A.* *verrilli*—28 tentacles, straight septa—doubtful species, sometimes considered a synonym of *A. wyvillei*; *A. valdiviae*—doubtful species, sometimes considered a synonym of *A. wyvillei*. ? refers to unknown status as we have not observed a specimen of *A. chuni*.

**Table A3:** Fixed differences for each *Atolla* species for *COI* barcode sequence (shaded in gray). Position refers to base pair position in alignment with *A. wyvillei* (GQ120088).

*COI* mtDNA	Position																										
Species	91	94	97	100	103	106	109	118	122	124	125	127	133	151	158	160	163	166	172	179	181	196	199	205	208	220	223
*Atolla* sp. B	G	T	T	T	C	A	A	T	T	A	T	A	A	T	A	A	A	A	T	C	A	A	T	A	T	T	A
*A. gigantea*	A	T	T	T	A	A	T	T	T	A	T	A	A	T	A	A	T	T	T	T	A	T	C	T	T	C	A
*A. reynoldsi*	A	G	C	C	T	C	C	C	C	T	G	G	T	A	G	G	T	T	A	T	A	G	G	G	C	A	G
*A. parva*	A	T	T	T	T	A	T	T	T	A	T	A	A	T	A	A	T	T	A	T	A	T	T	T	T	T	A
*A*. aff. *tenella*	A	T	T	T	G	A	T	T	T	A	T	A	A	T	A	A	T	T	T	T	G	T	T	T	T	C	A
*A. tenella*	A	T	T	T	T	A	T	T	T	A	C	A	A	T	A	A	C	T	G	T	A	T	T	T	T	T	A
*A. vanhoeffeni*	A	T	T	T	G	T	C	T	T	A	T	A	A	T	A	A	T	T	T	T	A	T	T	A	T	C	A
*A.* aff. *wyvillei*	A	T	T	T	T	A	T	T	T	A	T	A	A	T	A	A	T	T	A	T	A	T	T	T	T	T	A
*A. wyvillei*	A	T	T	T	A	A	T	T	T	A	T	A	A	T	A	A	C	T	T	T	A	T	C	T	T	C	A
***COI* cont.**	**Position**																										
Species	224	232	233	245	250	253	256	265	266	268	283	284	286	298	300	301	304	316	328	331	334	335	337	340	341	346	352
*Atolla* sp. B	T	T	A	T	A	A	C	A	T	A	A	T	A	A	C	C	T	T	C	A	T	G	T	A	T	T	T
*A. gigantea*	T	T	A	C	A	A	T	T	T	A	T	T	A	A	T	T	T	T	C	A	T	G	T	T	T	T	T
*A. reynoldsi*	T	C	G	T	G	T	T	C	C	T	G	A	T	G	T	T	A	A	T	G	T	G	T	T	T	T	A
*A. parva*	T	T	A	C	C	A	T	T	T	A	T	T	A	A	T	A	T	T	T	A	T	G	T	T	T	T	T
*A*. aff. *tenella*	T	T	A	C	C	A	T	T	T	A	T	T	A	A	T	A	T	T	C	A	T	G	T	C	C	T	T
*A. tenella*	T	T	A	C	T	A	T	T	T	A	T	T	A	A	T	A	G	T	T	A	C	G	C	T	T	A	T
*A. vanhoeffeni*	C	T	A	C	A	A	T	C	T	A	T	T	A	A	T	T	T	T	A	A	T	A	T	T	A	T	T
*A.* aff. *wyvillei*	T	T	A	C	C	A	T	T	T	A	T	T	A	A	T	G	T	T	T	A	T	G	T	T	T	T	T
*A. wyvillei*	T	T	A	C	A	A	T	T	T	A	T	T	A	A	T	A	T	T	C	A	T	G	C	T	T	T	T
***COI* cont.**	**Position**																										
Species	358	361	364	368	371	373	376	379	382	385	394	398	410	415	421	430	439	440	442	445	452	454	455	457	461	465	466
*Atolla* sp. B	A	T	A	T	A	A	A	T	T	T	T	T	T	T	C	T	T	A	A	T	A	C	A	A	C	T	T
*A. gigantea*	T	A	A	C	G	T	A	T	T	A	A	G	T	T	A	A	A	A	T	T	T	A	A	T	A	C	A
*A. reynoldsi*	T	A	G	T	G	C	T	C	T	A	A	C	C	A	A	C	T	G	G	C	G	T	C	T	C	C	A
*A. parva*	T	A	T	T	G	T	A	T	T	A	A	C	T	T	A	A	T	A	T	T	T	T	A	T	G	C	A
*A*. aff. *tenella*	T	A	A	T	G	C	A	T	T	A	A	G	T	T	A	A	A	A	A	T	T	A	A	T	G	C	A
*A. tenella*	T	A	T	T	G	T	A	T	T	A	A	A	T	T	A	A	C	A	A	T	T	T	A	T	G	C	A
*A. vanhoeffeni*	T	A	A	T	G	T	A	T	T	A	A	G	A	T	A	A	A	A	A	T	T	A	A	T	G	C	A
*A.* aff. *wyvillei*	T	A	T	T	G	T	A	T	C	A	A	C	T	T	A	A	T	A	A	T	T	T	A	T	G	C	A
*A. wyvillei*	T	A	A	T	G	T	A	T	T	A	A	G	T	T	A	A	A	A	T	T	T	A	A	T	A	C	A
***COI* cont.**	**Position**																										
Species	481	482	484	490	496	500	502	503	511	518	520	529	535	538	541	547	548	549	556	565	583	586	589	592	610	616	619
*Atolla* sp. B	A	T	A	T	T	T	A	G	T	T	A	T	T	G	A	A	G	C	T	A	T	T	A	T	G	T	A
*A. gigantea*	T	T	A	G	A	T	A	G	T	C	T	T	A	A	A	A	G	C	A	A	T	T	C	T	A	T	T
*A. reynoldsi*	T	C	T	G	C	C	G	G	A	C	T	A	G	A	G	A	T	G	A	G	C	C	G	C	C	T	T
*A. parva*	C	T	A	A	T	T	A	G	T	C	T	T	A	T	A	G	G	C	A	A	T	T	T	T	T	T	T
*A*. aff. *tenella*	T	T	A	A	A	T	A	G	T	C	T	T	G	A	A	A	G	C	A	A	T	T	T	T	A	C	T
*A. tenella*	T	T	A	A	T	T	A	G	T	C	T	T	A	T	A	A	G	C	A	A	T	T	T	T	T	T	T
*A. vanhoeffeni*	T	T	A	A	A	T	A	A	C	C	T	T	A	A	A	A	G	C	A	A	T	T	T	T	G	T	T
*A.* aff. *wyvillei*	C	T	A	A	T	T	A	G	T	C	T	T	A	T	A	A	G	C	A	A	T	T	T	T	T	T	T
*A. wyvillei*	T	T	A	A	T	T	A	G	T	C	T	T	A	A	A	A	G	C	A	A	T	T	C	T	A	T	T
***COI* cont.**	**Position**																										
Species	640	646	652	664	670	671	673	676	677	685	694	695	697	700	712	718	721	724	760	763	769	778	779	781			
*Atolla* sp. B	T	T	G	A	T	T	A	A	C	T	A	A	A	A	T	T	T	T	T	G	G	T	T	A			
*A.gigantea*	T	T	A	A	T	T	A	T	T	T	A	G	A	T	A	T	T	T	T	T	T	T	A	A			
*A. reynoldsi*	C	C	T	G	T	C	T	T	T	C	G	G	T	T	A	C	T	T	C	T	A	C	C	T			
*A. parva*	T	T	C	T	T	T	A	T	T	T	A	G	G	T	A	T	T	T	T	T	T	T	A	C			
*A*. aff. *tenella*	T	T	C	T	T	T	A	T	T	T	A	G	A	T	A	T	C	C	T	T	T	T	A	A			
*A. tenella*	T	T	C	C	C	T	G	T	T	T	A	G	A	T	A	T	T	T	T	T	T	T	A	A			
*A. vanhoeffeni*	T	T	T	T	T	T	A	T	T	T	A	G	A	T	A	T	T	T	T	T	T	T	A	A			
*A.* aff *wyvillei*	T	T	C	T	T	T	A	T	T	T	A	G	A	T	A	T	T	T	T	C	T	T	A	C			
*A. wyvillei*	T	T	A	C	T	T	A	T	T	T	A	G	A	T	A	T	T	T	T	T	T	T	A	A			
